# Quantitative Imaging of D-2-Hydroxyglutarate in Selected Histological Tissue Areas by a Novel Bioluminescence Technique

**DOI:** 10.3389/fonc.2016.00046

**Published:** 2016-03-07

**Authors:** Nadine F. Voelxen, Stefan Walenta, Martin Proescholdt, Katja Dettmer, Stefan Pusch, Wolfgang Mueller-Klieser

**Affiliations:** ^1^Institute of Pathophysiology, University Medical Center of the Johannes Gutenberg University Mainz, Mainz, Germany; ^2^Department of Neurosurgery, University Hospital Regensburg, Regensburg, Germany; ^3^Institute of Functional Genomics, University of Regensburg, Regensburg, Germany; ^4^Clinical Cooperation Unit Neuropathology, German Cancer Research Center (DKFZ), Heidelberg, Germany

**Keywords:** D-2 hydroxyglutarate, IDH mutations, bioluminescence imaging, oncometabolite, glioblastoma

## Abstract

Patients with malignant gliomas have a poor prognosis with average survival of less than 1 year. Whereas in other tumor entities the characteristics of tumor metabolism are successfully used for therapeutic approaches, such developments are very rare in brain tumors, notably in gliomas. One metabolic feature characteristic of gliomas, in particular diffuse astrocytomas and oligodendroglial tumors, is the variable content of D-2-hydroxyglutarate (D2HG), a metabolite that was discovered first in this tumor entity. D2HG is generated in large amounts due to various “gain-of-function” mutations in the isocitrate dehydrogenases *IDH1* and *IDH2*. Meanwhile, D2HG has been detected in several other tumor entities, including intrahepatic bile-duct cancer, chondrosarcoma, acute myeloid leukemia, and angioimmunoblastic T-cell lymphoma. D2HG is barely detectable in healthy tissue (<0.1 mM), but its concentration increases up to 35 mM in malignant tumor tissues. Consequently, the “oncometabolite” D2HG has gained increasing interest in the field of tumor metabolism. To facilitate its quantitative measurement without loss of spatial resolution at a microscopical level, we have developed a novel bioluminescence assay for determining D2HG in sections of snap-frozen tissue. The assay was verified independently by photometric tests and liquid chromatography/mass spectrometry. The novel technique allows the microscopically resolved determination of D2HG in a concentration range of 0–10 μmol/g tissue (wet weight). In combination with the already established bioluminescence imaging techniques for ATP, glucose, pyruvate, and lactate, the novel D2HG assay enables a comparative characterization of the metabolic profile of individual tumors in a further dimension.

## Introduction

Heterozygous mutations in catalytic arginine residues of isocitrate dehydrogenases (IDHs) 1 and 2 (*IDH1* and *IDH2*) have been identified during exome-sequencing studies of glioblastoma tumors in 2008 (1). These mutations can be found in approximately 75% of diffuse astrocytoma and oligodendroglioma tumors (2–4). Moreover, in 20% of acute myeloid leukemia (AML) (5, 6), 50% of chondrosarcoma (7, 8), 20% of intrahepatic cholangiocarcinoma (9), and 20% of angioimmunoblastic T-cell lymphoma, *IDH1* or *IDH2* are mutated as well (10). Regardless of the overall frequency, the type of *IDH* mutations differs in the described tumor entities: in astrocytoma and oligodendroglioma, more than 90% of all *IDH* mutations are of the *IDH1R132H* type (3), whereas the second most frequent type (about 4% of mutations) is *IDH1R132C*. It has been suggested recently that natural selection may act against the rare *IDH1R132* mutations in human glioma due to the cytotoxicity of high levels of D-2-hydroxyglutarate (D2HG) (11). Malignancies other than gliomas show different mutation spectra. In AML, the most frequent mutation is *IDH2R140Q* (6, 12); while in chondrosarcoma and intrahepatic cholangiocarcinoma, *IDH1R132C* represents the most frequent mutation (7, 9). In angioimmunoblastic T-cell lymphoma, mutations are found most frequently in *IDH2* (10). 2-HG has been described initially in the context of hereditary 2-HG aciduria in 1980. 2-HG aciduria is caused by germline loss-of-function mutations in either D-2-hydroxyglutarate dehydrogenase (D2-HGDH) or L-2-hydroxyglutarate dehydrogenase (L2-HGDH) (13–17).

**Figure 1:**
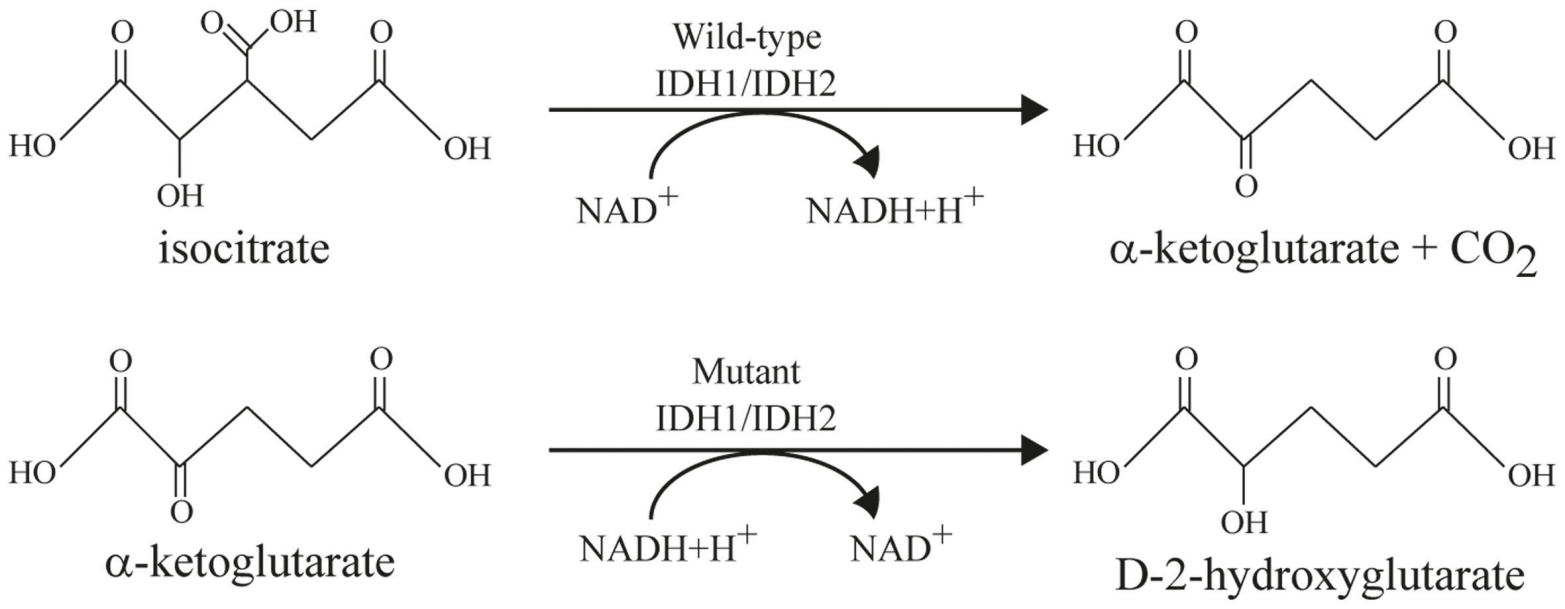
**Enzymatic reactions catalyzed by wild-type and mutant IDH enzymes modified after Cairns and Mak (21)**. *Top line*: wild-type IDH1 and IDH2 catalyzes the oxidative decarboxylation of isocitrate to α-ketoglutarate and CO_2_ while converting NAD^+^ to NADH + H^+^. *Bottom line*: mutant IDH1 and IDH2 reduce α-ketoglutarate to D-2-hydroxyglutarate (D2HG) while converting NADH + H^+^ to NAD^+^.

Interest in D-2-hydroxyglutarate (D2HG) as an “oncometabolite” has gained momentum only recently. Tissue concentrations of D2HG, which is hardly detected in normal tissue (<0.1 mM), as high as 35 mM have been reported for malignant tumors (18). Such an extraordinary difference in metabolite concentration between tumor and normal tissues has been described previously only for lactate (19). At first, D2HG had been described solely in glioblastomas and AML. In the meantime, it has been detected in additional tumor entities, such as intrahepatic bile-duct cancer, and chondrosarcoma (20). The first review articles about D2HG suggested a common importance and role of this “oncometabolite” in tumor progression of different entities (21–23). Elevated D2HG levels are produced in tumor cells that contain a “gain-of-function” mutation of the IDH (21–23). From the three known isoforms of IDH, only somatic mutations in *IDH1* and *IDH2* have been reported to date to contribute to tumorigenesis. The IDH-gene accounts for the most frequently mutated metabolic gene of all human tumors (24). As depicted in Figure 1, the wild-type enzyme (wild-type IDH1/IDH2) generates alpha-ketoglutarate (α-KG; also referred to as 2-oxoglutarate, 2-OG) while reducing NADP^+^ to NADPH + H^+^ and liberating CO_2_. The mutated enzyme (mutant IDH1/IDH2), in contrast generates D2HG while consuming NADPH. IDH1 and IDH2 are highly homologous, but they are distinct from the NAD^+^-dependent, heterotetrameric IDH3 enzyme that functions in the tricarboxylic acid (TCA) cycle. The physiological role of the NAD^+^-dependent IDH1/2 enzymes is not well characterized yet, but they are assumed to play different roles in the metabolism of glucose, fatty acids, glutamine, and to contribute to the maintenance of normal cellular redox status (21, 25). *In vitro* analyses indicate that cell membranes are impermeable for D2HG and the metabolite is barely taken up by cells in culture, compromising the direct investigation of the effects of *IDH* mutations and D2HG in cell culture systems (26). As a consequence, the function of D2HG is poorly understood.

The chiral molecule is very similar in its structure to the achiral 2-OG and is normally present at low levels in either its D- (or R-) enantiomeric forms. Recently, it has been demonstrated that hypoxia induces selectively the production of the enantiomer L-2-hydroxyglutarate (L2HG) in mammalian cells due to promiscuous substrate usage primarily by lactate dehydrogenase A (27). However, *in vitro* and *in vivo* experiments have unraveled that the mutated IDH enzymes exclusively produce the D-enantiomeric form of 2-HG. These findings combined with the pattern of mutations in these genes support the concept that the production of D2HG by the mutant enzyme is responsible for driving tumor progression. Hence, D2HG has been described as an “oncometabolite” in glioma and AML (21). The molecular mechanisms by which D2HG promotes tumorigenesis are not yet fully understood, but competitive inhibition of 2-OG-dependent dioxygenases, such as the DNA-modifying enzymes TET (Ten-eleven translocation methylcytosine dioxygenase) and histone-demethylases of the JmjC-type appears to play an important role (23). Furthermore, the recent observation that 2HG is capable of inhibiting ATP synthase and mTOR signaling and thus, tumor cell death under conditions of glucose limitations has provided a basis for understanding the better prognosis of IDH-mutant brain tumors (28).

It has been suggested that an elevated intratumoral level of 2HG might serve as a suitable surrogate marker for the non-invasive detection of *IDH* mutations by magnetic resonance spectroscopy (29). In body fluids such as plasma and urine and tissue extracts, total 2HG content has also been determined by liquid chromatography/mass spectrometry (LC/MS), gas chromatography/mass spectrometry (GC/MS) (29–31), and ^13^C NMR spectroscopy (32). Combined with appropriate chiral selectors or chiral derivatizing agents, the aforementioned methods may also be used to determine the concentrations of the individual enantiomers (16, 33, 34). Furthermore, the stereospecificity of enzymes may be exploited for the selective determination of 2HG enantiomers, which carries the additional advantages of high-throughput and low instrumental cost (35). However, these methods require extraction of 2HG from tissues, whereby critical information with regard to the cellular source and potential tissue gradients of 2HG or its individual enantiomers is lost.

The aim of this study was to exploit the versatility of the bioluminescence technique currently used in our lab for the determination of ATP, glucose, pyruvate, and lactate for quantita­tive D2HG imaging whereas serial sectioning and precise overlay of sections allows for signal acquisition in selected histological areas such as tumor tissue versus surrounding connective tissue or epithelium. The induced metabolic bioluminescence imaging (imBI) technique established in our laboratory uses specific enzymes as biochemical probes for the detection of metabolites. This specific biochemical reaction is coupled quantitatively via an enzyme chain to the light emission of bacterial luciferases (bioluminescence), thus, providing the necessary sensitivity to detect micromolar amounts of metabolite (36). For further information on the imBI technique refer to Sattler et al. (37), Walenta et al. (38), Walenta et al. (39), and Walenta et al. (36).

## Materials and Methods

### Assessment of Specimens

With approval by the Ethics Committee of the University Medical Center Regensburg, Germany, and written informed consent by the patients, two frozen biopsies of human glioblastoma multiforme were provided by the Department of Neurosurgery at the University Hospital Regensburg. The frozen tissues were retrieved from liquid nitrogen and stored in an −80°C freezer for a minimum of 24 h. Crucial for obtaining accurate results is the immediate immersion of the biopsy specimens in liquid nitrogen. To prepare the biopsies for the imBi technique, tumor biopsies were sectioned at −20°C in a cryomicrotome into serial sections of 16 μm each for the detection of ATP, lactate, and glucose, and into sections of 20 μm for the detection of D2HG. The cryosections were then collected on cover slips and heated to 100°C for 10 min to inactivate endogenous enzymes that might confound the measurements.

### D2HG-Dependent Bioluminescence

#### Enzyme Solution

For the measurement of D2HG, we made use of the *D2HGDH* gene from *Acidaminococcus fermentans* (40), which had been previously cloned into a pASK-IBA7plus expression plasmid, expressed in *E. coli* and purified using the Strep^®^-tag affinity chromatography system (35). The constituents of the enzyme solution for D2HG-dependent bioluminescence measurements are listed in Table 1. For further information concerning expression and purification of D2-HGDH, see Balss et al. (35).

**Table 1:** **Constituents of the enzyme solution employed for the quantitative determination of D2HG in cryosections using induced metabolic bioluminescence imaging (imBI)**.

Constituent	Concentration	Manufacturer
Phosphate buffer solution (containing KH_2_PO_4_ and Na_2_HPO_4_) (pH 7.0 at 20°C)	0.1 M	Applichem, Darmstadt, Germany; Sigma-Aldrich, St.Louis, MO, USA
Dithiothreitol (DTT)	0.4 mM	Sigma-Aldrich, St.Louis, MO, USA
Nicotinamide adenine dinucleotide (NAD) (free-acid grade)	20 mM	Roche Diagnostics GmbH, Mannheim, Germany
Flavin mononucleotide (FMN)	0.25 M	Applichem, Darmstadt, Germany
Decanal dissolved in methanol	6 mM	Merck KGaA, Darmstadt, Germany; VWR international, Darmstadt, Germany
NAD(P)H-FMN-oxidoreductase (NFO)	3.6 units/mL	Roche Diagnostics GmbH, Mannheim, Germany
Luciferase from *Photobacterium fischeri*	13.3 units/mL	Roche Diagnostics GmbH, Mannheim, Germany
Hydroxyglutarate dehydrogenase (HGDH)	5.52 μg/μL	kindly provided by S.Pusch from the lab of A.v.Deimling, DKFZ, Heidelberg, Germany

#### Calibration Standards

Standards of D2HG were obtained by dissolving D-2-hydroxyglutaric acid disodium salt (Sigma-Aldrich, St Louis, MO, USA) in 50 mM MOPS-Cl buffer pH 7.4 (Sigma-Aldrich, St Louis, MO, USA). Then, an aliquot of this D-2-hydroxyglutaric acid MOPS-Cl solution was mashed with three parts of Tissue-Tek^®^ *O.C.T*. Compound (Sakura Finetek Europe B.V., Alphen aan den Rije, Netherlands) (4:1), mixed slowly for 30 min and afterwards frozen at −20°C until sectioning using a cryomicrotome. The section thickness was 16 or 20 μm. Standards used in former published studies using imBI were obtained from tissue homogenates (37) and the analysis of these homogenates revealed the same results as the usage of Tissue Tek OCT and, therefore, Tissue Tek OCT compound was used.

#### Bioluminescence Measurement Procedure

For the bioluminescence imaging measurements, a cover glass containing a standard section with a known concentration or a section from an actual biopsy was placed upside down on a metal slide containing a casting mold filled with an excess amount (50 μL) of the above-mentioned enzyme solution. Thereby, the contact between the tissue sample and the enzyme solution initiates the bioluminescence reaction. The “reaction sandwich” was immediately placed on a thermostated stage of a microscope (Axiophot, Zeiss, Oberkochen, Germany) within a dark chamber. After 40 s of incubation time at 20°C, the bioluminescence was registered for a defined interval of time, determined by a precedent kinetic control measurement. The emission of light was detected by a long distance precision lens (magnification 1.25×; Zeiss, Oberkochen, Germany) associated with a circular viewing field of 10 mm in diameter. A back-illuminated EM-CCD camera (iXonEM + DU-888; Andor Technology PLC, Belfast, UK) connected to the microscope enables the registration of the low light bioluminescence intensity. The image signal is transferred to a computer for further analysis. By integration of the light emission intensities over a selected time frame, it is possible to obtain a density profile that represents the metabolite distribution across the tissue section analyzed. The measured profiles are calibrated in micromole of metabolite/gram of tissue (micromolar/gram; equivalent to millimolar/liter or millimolar in solution) and are displayed in a color-coded way. Based on the high water content of tumor cells of between 80 and 93% (41), 1 g of tumor tissue may be approximated by a volume of 1 mL. If we assume that this is approximately the volume of the solute for D2HG or other metabolites, a respective concentration of 1 μmol/mL or 1 mmol/L (millimolar) is obtained. Serial sectioning and precise overlay of sections allows for signal acquisition in selected histological areas, such as in tumor tissue versus surrounding connective tissue or epithelium. For further methodological details, see Mueller-Klieser and Walenta (42), Sattler et al. (37), Walenta et al. (43), and Walenta et al. (36).

#### Image Analysis

The bioluminescence images of standard samples were analyzed for their mean light intensity using Andor iQ imaging software *(*Andor Technology PLC, Belfast, UK) and Excel (Microsoft, Redmond, WA, USA). After measuring the standard samples containing different amounts of D2HG, a calibration curve was generated that allowed the illustration of the two-dimensional substrate distribution of D2HG in tissue samples as well. The bioluminescence images obtained from tumor sections were transferred as 8-bit and 16-bit images to Adobe Photoshop CS5 software (Adobe Systems Incorporated, San Jose, CA, USA), ImageJ (National Institute of Health, Bethesda, MD, USA), and Excel (Microsoft, Redmond, WA, USA). Using Photoshop software the bioluminescence images from tumor sections were overlaid with images of sequential sections stained with hematoxylin (Carl Roth GmbH, Karlsruhe, Germany) and eosine (Merck KGaA, Darmstadt, Germany) (HE) to analyze the data within selected histological areas, such as viable tumor regions, necrosis, or tumor-adjacent normal tissue. After overlaying the tissue samples, evaluation masks were drawn using a brush tool and a drawing-pad (BambooPad, Wacom Europe GmbH, Krefeld, Germany) in Adobe Photoshop CS5 imaging software. These evaluation masks were subsequentially loaded in ImageJ software and analyzed based on a semi-automated analysis method (Plug-in) called “tumor statistics,” which transfers the exact position and size of the evaluation mask to the 16-bit bioluminescence image and estimates the mean light intensity within this area and its SD automatically. The obtained data were transferred to Excel and OriginPro 8G Software (OriginLab Corporation, Northampton, MA, USA) for further statistical analysis. Representative images were calibrated in micromole of metabolite/gram of tissue in micromolar/gram (wet weight; equivalent to millimolar/liter in solution) in a color-coded manner. Using appropriate standards, which are handled in exactly the same way as the tissue of interest, bioluminescence intensities can be transformed into absolute tissue concentrations of the respective metabolite, e.g., in micromoles/gram of tissue (micromolar/gram), which corresponds approximately to millimolar in solution. Additionally, while preparing the standard samples for calibration, the weights of the D2HG solution and the Tissue-Tek OCT compound are determined with an analytical balance to identify the actual concentration of the D2HG solution.

### Fluorimetric D2HG Assay

For the fluorimetric determination of D2HG, we used a slightly modified assay previously established by the lab of Andreas von Deimling at the DKFZ in Heidelberg, Germany (35). With the help of the expressed and purified D2HGDH enzyme, Balss et al. had previously developed a fluorimetric assay, in which D2HGDH catalyzes the oxidation of D2HG to α-KG generating NADH + H^+^ in the process. The hydrogen from the accumulated NADH is then transferred by diaphorase to the non-fluorescent resazurin resulting in the production of fluorescent resorufin (44). The fluorimetric detection was performed with excitation and emission wavelengths of 540 ± 10 nm and 610 ± 10 nm, respectively, on a multiplate reader (35). This assay showed high accordance with LC/MS measurements. Therefore, we set up a standard series of 0–50 μM of D2HG and treated the standard samples (*n* = 5 for each concentration) under two different conditions: one standard row was treated for 1 min with sonication and was heat-autoclaved for 10 min. The other row was left untreated. The constituents of the D2HG-based fluorimetric solution are listed in Table 2. The standards and samples were analyzed immediately after excessive mixing at room temperature using the fluorimetric setting in the multiplate reader DTX 880 (Beckman Coulter, Krefeld, Germany). Afterwards, a calibration curve was generated applying appropriate Excel and Origin 8G software.

**Table 2:** **Constituents of the enzyme solution employed for the quantitative determination of D2HG in solution by fluorimetrical analysis**.

Constituent	Concentration	Manufacturer
HEPES buffer pH 8.0	100 mM	Sigma-Aldrich, St.Louis, MO, USA
Nicotinamide adenine dinucleotide (NAD) (free-acid grade)	100 μM	Roche Diagnostics GmbH, Mannheim, Germany
Diaphorase	0.1 units/μL	MP Biomedicals, Heidelberg, Germany
Resazurin	5 μM	Applichem, Darmstadt, Germany
Hydroxyglutarate dehydrogenase (HGDH)	5.52 μg/μL	kindly provided by S.Pusch from the lab of A.v.Deimling, DKFZ, Heidelberg, Germany

### Determination of 2HG by LC-MS/MS

Frozen tissue samples (#1385: 32.7 mg, #1586: 58.0 mg) were transferred to “Precellys-Keramik-Kit 1.4 mm” vials followed by 20 μL stable isotope-labeled internal standard solution (2,3,3-d3-2HG in water, 100 μM) and 1000 μL 80% methanol (MeOH:H2O, 80:20, v/v). The samples were homogenized twice at 6500 rpm for 20 s with an intermediate pause of 15 s using a Precellys homogenizer (Peqlab Biotechnologie GmbH, Erlangen, Germany). The homogenate was centrifuged at 9900 × *g* for 5 min at 4°C and the supernatant was transferred to a 1.5-mL glass vial. The pellet was washed consecutively with 500 and 300 μL 80% methanol followed by a final wash with 300 μL water. All supernatants from one sample were combined and evaporated using an infrared vortex vacuum evaporator (CombiDancer, Hettich AG, Baech, Switzerland). The residues were reconstituted in 100 μL water and analyzed by HPLC-ESI-MS/MS using an Agilent 1200 SL HPLC (Boeblingen, Germany) and a 4000 QTrap mass spectrometer (AB SCIEX, Darmstadt, Germany). A Discovery HS F5-3 HPLC column (15 cm × 2.1 mm, 3 μm; Supleco, Bellefonte, PA, USA) equipped with a Security Guard column (C18, Phenomenex, Aschaffenburg, Germany) was used with mobile phase A consisting of 0.1% formic acid in water (v/v) and acetonitrile as mobile phase B. Gradient elution started with an isocratic hold at 0% B for 6.5 min and a flow rate of 200 μL/min followed by a linear increase to 100% B in 1.5 min using a flow rate of 350 μL/min, which was held for 2 min. For equilibration, the solvent was changed back to 0% B from 10 min to 10.1 min and held until 17 min. The flow rate was reduced back to 200 μL/min at 17.1 min and equilibrated for 1.9 min. The column was kept at 30°C, and an injection volume of 5 μL was used. The mass spectrometer was operated in negative mode using turbo ion spray employing the following parameters: gas 1 and 2: 50 and curtain gas: 10 (arbitrary units).The ion spray voltage was set to −4500 V, the declustering potential to −40.0 V, the entrance potential to −10.0 V, the collision exit potential to −5 V, and the collision energy to −24 V. Detection was performed in multiple reaction monitoring (MRM) mode using the following ion transitions: *m/z* 147.1 (M + H)+ to *m/z* 84.8 for 2HG and *m/z* 150.1 to *m/z* 87.8 for the deuterated internal standard. Quantification was achieved using a calibration curve with the area of 2HG normalized by the area of the stable isotope-labeled standard (deuterated 2HG).

### Statistics

Data were presented as the mean ± SD. For statistical analysis, OriginPro 8G Software (OriginLab Corporation, Northampton, MA, USA) and Microsoft Office Excel (Microsoft, Redmond, WA, USA) were used to perform Box Lucas equation for a non-linear regression curve and Student’s *t*-tests. For descriptive analysis, results were shown in boxplots (black bar in the middle: mean; boxes: 25 and 75% percentile; whiskers: SD). A *p*-value <0.05 was considered as being statistically significant.

## Results

### Stability of D-2-Hydroxyglutarate

**Figure 2:**
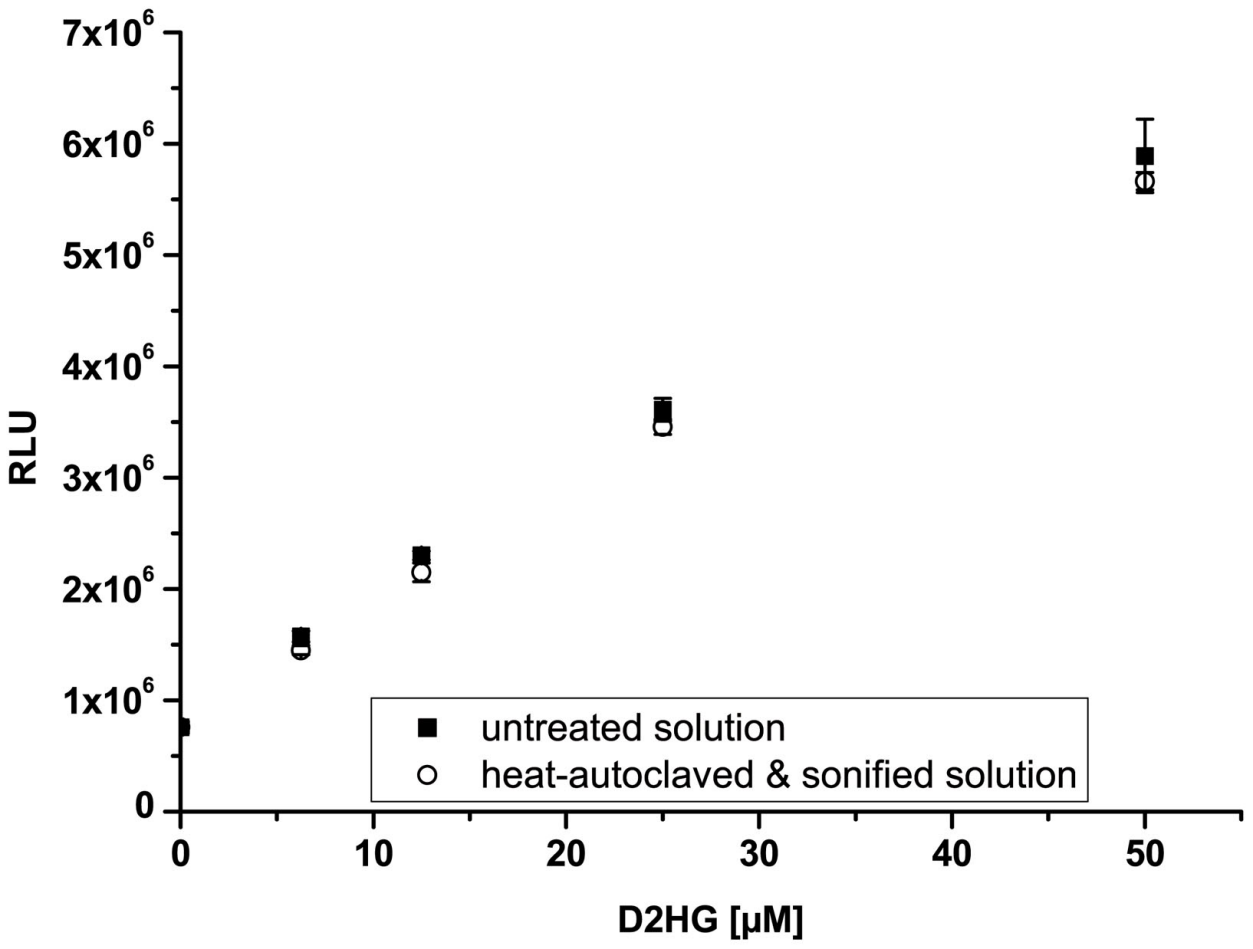
**Stability of D-2-hydroxyglutarate (D2HG)**. Relative light units (RLU) as a function of D2HG concentration assayed photometrically via fluorescence assay. Mean values from quintuple measurements ± SD are shown for untreated *(closed squares)* and heat and mechanical treated *(open circles)* D2HG solutions. (Linear regression: *y* = 97767*x* + 863877; *R*^2^ = 0.997; *n* = 5; *p* < 0.00001).

It was necessary to test the chemical stability of D2HG, since tissue sections have to be heated to inactivate endogenous enzymes and to fix them to the cover glass before bioluminescence imaging. In addition, the stability of D2HG values in tissue was tested by sonication of the standard samples. D2HG was, therefore, tested photometrically in aqueous solutions by the fluorimetric D2HG assay established by Balss et al. (35), following heat or sonication compared to untreated control samples. Figure 2 shows that heat-autoclaving and sonication of D2HG solution has no degrading effect compared to untreated control samples (*n* = 5) and still revealed a concentration-dependent increase in relative light units (RLU) up to a D2HG concentration of 50 μM.

### Biochemical Principle of D2HG-Dependent Bioluminescence

**Figure 3:**
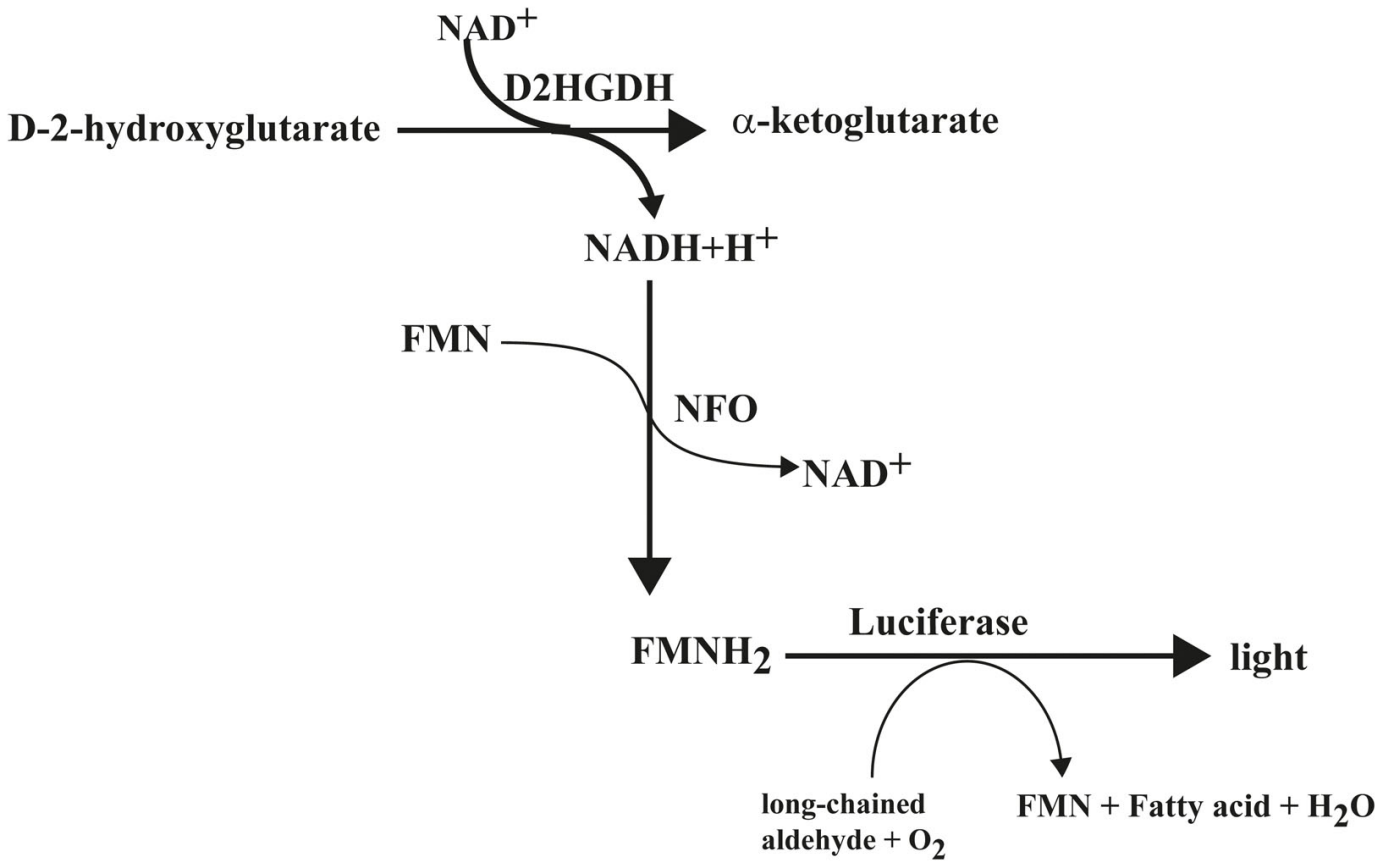
**Biochemical reaction scheme of D-2-hydroxyglutarate-dependent bioluminescence**. D2HGDH catalyzes the oxidation of D-2-hydroxyglutarate to α-ketoglutarate, while converting NAD^+^ to NADH + H^+^, which is linked to a light reaction via NFO and bacterial luciferase. Abbreviations: D2HGDH, D-2-hydroxyglutarate dehydrogenase; NFO, NADH:FMN-oxidoreductase.

Biochemically, the principle of D2HG-dependent bioluminescence is based on the luminescence of luciferase from *Photobacterium fischeri*. The substrate is first linked to the Nicotinamide adenine dinucleotide (NAD)/NADH + H^+^ redox system via the specific enzyme. Next, the redox system is connected to the bacterial luciferase *Photobacterium fischeri* through flavin mononucleotide (FMN). In principle, it is possible to measure any substance that can be linked quantitatively to NAD/NADH + H^+^. Therefore, this fact was utilized in the present study to analyze the concentration and regional distribution of D2HG in cryosections. The reactions involved in the detection of D2HG are depicted in Figure 3. D-2-hydroxyglutarate (D2HG) is converted via the specific enzyme D-2-hydroxyglutarate-dehydrogenase (D2HGDH) into Alpha-ketoglutarate under the consumption of NAD^+^. The generated NADH is then linked to the light reaction by the enzyme NADH:FMN-oxidoreductase (NFO), which converts NADH + H^+^ and FMN to FMNH_2_. The luciferase from *Photobacterium fischeri*, which is present in the mixture as well finally catalyzes the bioluminescent oxidation of FMNH_2_ and a long-chained aliphatic aldehyde. The aldehydes necessary for this reaction are long-chained fatty aldehydes. In the case of Luciferase from *Photobacterium fischeri*, as used in our assay, the long-chained fatty aldehyde is palmitic aldehyde (1-hexadecanal; C16H32O). In our assay, we use 5.69 mM decanal (C9H19CHO; caprinaldehyde) dissolved in methanol as long-chained aldehyde as substrate for the luciferase reaction. The light emission during this process is proportional to the initial D2HG concentration.

### D2HG-Dependent Bioluminescence

**Figure 4:**
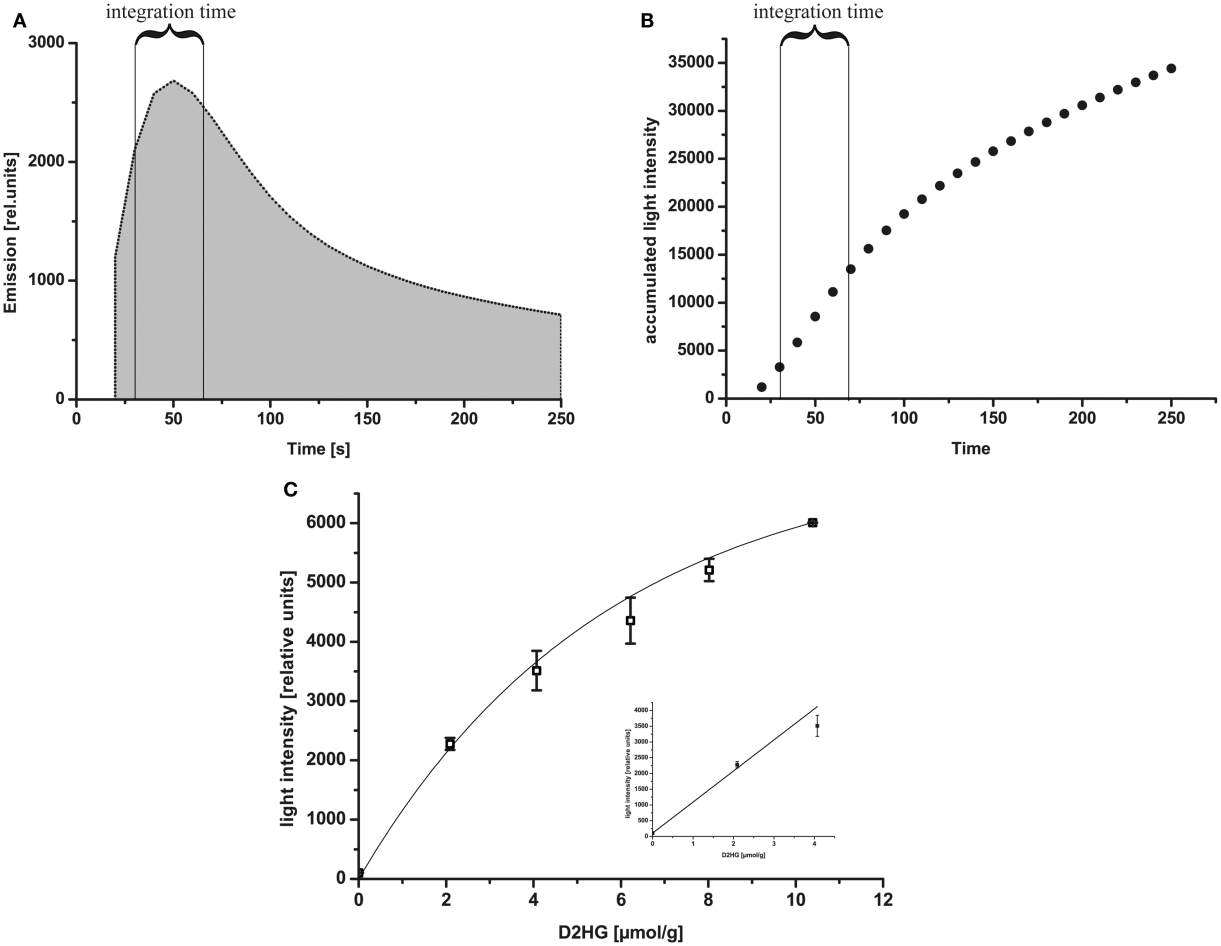
**(A)** Reaction kinetics of D2HG-dependent bioluminescence. Relative emission intensity of the D2HG-dependent bioluminescence mixture within 4 min. A 10 μmol/g standard section (20 μm) was used for measurement based on the D2HG enzyme mixture. The integration time is indicated in the figure. **(B)** Accumulated light intensities obtained from D2HG kinetics. The integration time is indicated in the figure. **(C)** D-2-hydroxyglutarate-dependent bioluminescence using D-2-hydroxyglutaric acid standard solutions mashed with Tissue-Tek^®^ *O.C.T*. embedding medium at a temperature of 20°C. Representative calibration curve of D2HG-dependent bioluminescence from D-2-hydroxyglutaric acid standards: light intensity ± SD using 20 μm sections of frozen D-2-hydroxyglutaric acid standards (relative units) as a function of D2HG concentrations in micromolar/gram of tissue (wet weight; equivalent to mmol/L). BoxLucas: *y* = 7160*(1−0.83*x*); *r*^2^ = 0.768; *p* < 0.05; *n* = 3 for all concentrations. Insert plot displays the D2HG concentrations plotted against the light intensities in the range between 0 and 4 μmol/g (linear regression: *y* = 987.09*x* + 104.62; *r*^2^ = 0.984; *p* < 0.05; *n* = 3 for all concentrations).

**Figure 5:**
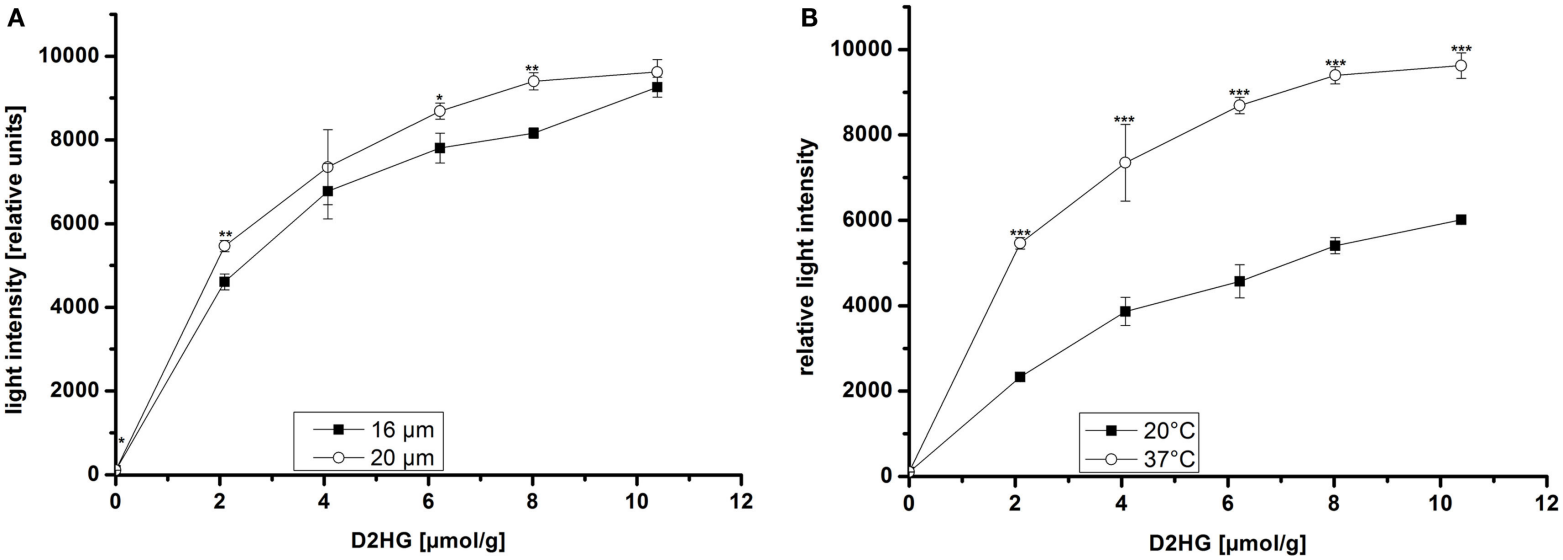
**Bioluminescence intensity as a function of (A) section thickness and (B) temperature of the measurement chamber at different D2HG concentrations**. Mean values from triplicate measurements ± SD are shown. Significant differences obtained by Student’s *t*-test are indicated by stars (*); *p* ≤ 0.05 (*), *p* ≤ 0.001 (***).

Via bioluminescence imaging (imBI) of standard samples containing a known amount of D2HG from 0 mM to 10 mM in solution (equivalent to micromolar/gram wet weight of tissue), a calibration curve was generated (Figure 4C). Using a non-linear regression curve after the box/lucas model through the data points of the calibration curve allows the conversion of measured light intensities from unknown samples into absolute concentrations in micromolar D2HG/gram tissue (wet weight) (BoxLucas: *y* = 7160*(1−0.83^x^); *r*^2^ = 0.768). As depicted in the inlay plot of Figure 4C regarding the D2HG concentrations between 0 and 4 μmol/g, there was a linear regression detectable between the D2HG concentrations and the relative light intensities (Linear regression: *y* = 987.09x + 104.62; *r*^2^ = 0.984). The light emission was registered within a defined time interval determined by the reaction kinetics (Figure 4A). The reaction kinetics were obtained from recordings of a 10 mM D2HG standard section every 10 s for a period of about 4 min. The means of light intensities received from the camera software of every 10-s time slot are plotted against time. In this way, it is possible to obtain the reaction kinetics from D2HG imBI and to define the time slots for incubation and measurement resulting in a protocol for the measurements of the standard calibration curve and the measurements of actual tissue biopsies. The area around the highest peak of light intensities of the reaction kinetics constitutes the time slot used for imBI measurement. The time until reaching the highest peak of light intensity is thereby standardized and routinely used as incubation and light integration time. Additionally, the reaction kinetics can be visualized more clearly using the accumulated light intensities obtained from the D2HG kinetics (see Figure 4B). Furthermore, it could be shown that the light intensity of imBI measurements was dependent on the thickness of the tissue sections as well as the temperature in the measurement chamber (Figure 5). Increasing the thickness of sections to 20 μm resulted in a significant augmentation in light intensity of the standard curve at 2, 6, and 8 μmol/g D2HG, compared to the light intensity of 16 μm tumor sections (Figure 5A). Heating of the measurement chamber to 37°C resulted in a highly significant increase in light intensity at 2, 4, 6, 8, and 10 μmol/g D2HG, compared to the light intensity at 20°C (Figure 5B). We conclude from the data shown in Figure 5 that by keeping section thickness and temperature constant, the light intensity generated by the D2HG bioluminescence assay is consistently proportional to the initial D2HG concentration, as prepared for the standard sections. The assay makes it possible to determine D2HG concentrations in frozen tissue sections in the range of 0–10 μmol/g of tissue (wet weight).

### Quantification of D2HG in Tumor Biopsies of Human Glioblastoma Multiforme with Unknown IDH Mutation Status

**Figure 6:**
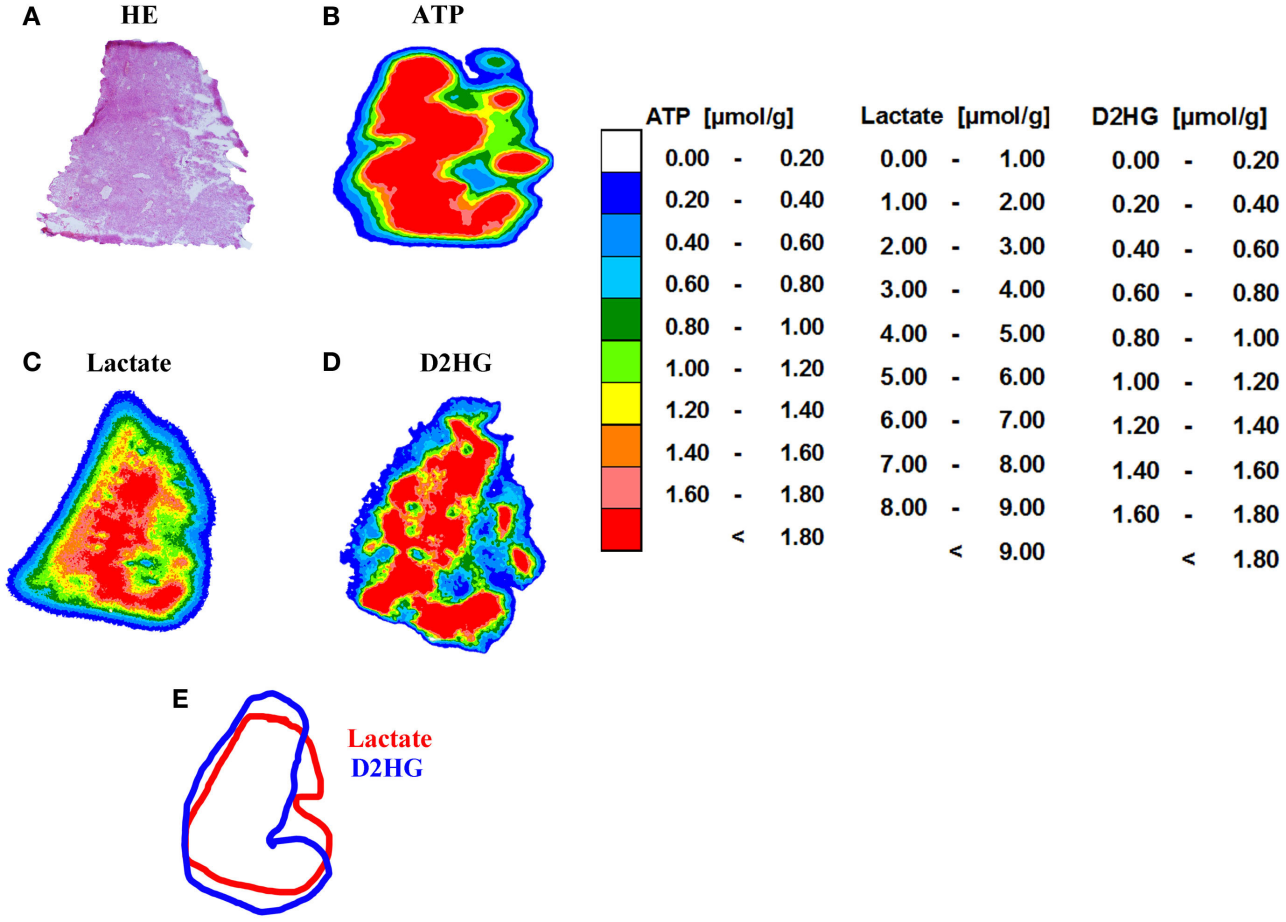
**Imaging bioluminescence of ATP, lactate, and D2HG in a human WHO grade IV glioblastoma**. **(A)** Hematoxylin and eosin (H&E) staining (10 μm) as well as color-coded distributions of concentrations of **(B)** ATP (16 μm); **(C)** lactate (16 μm), and **(D)** D-2-hydroxyglutarate (20 μm). There is an obvious similarity in the distribution pattern of lactate and D2HG. **(E)** Regional overlay of the analysis masks for lactate and D2HG. *Tissue biopsies were kindly provided by Prof. Alf Giese from the Department of Neurosurgery, University Medical Center Mainz, Germany*.

**Figure 7:**
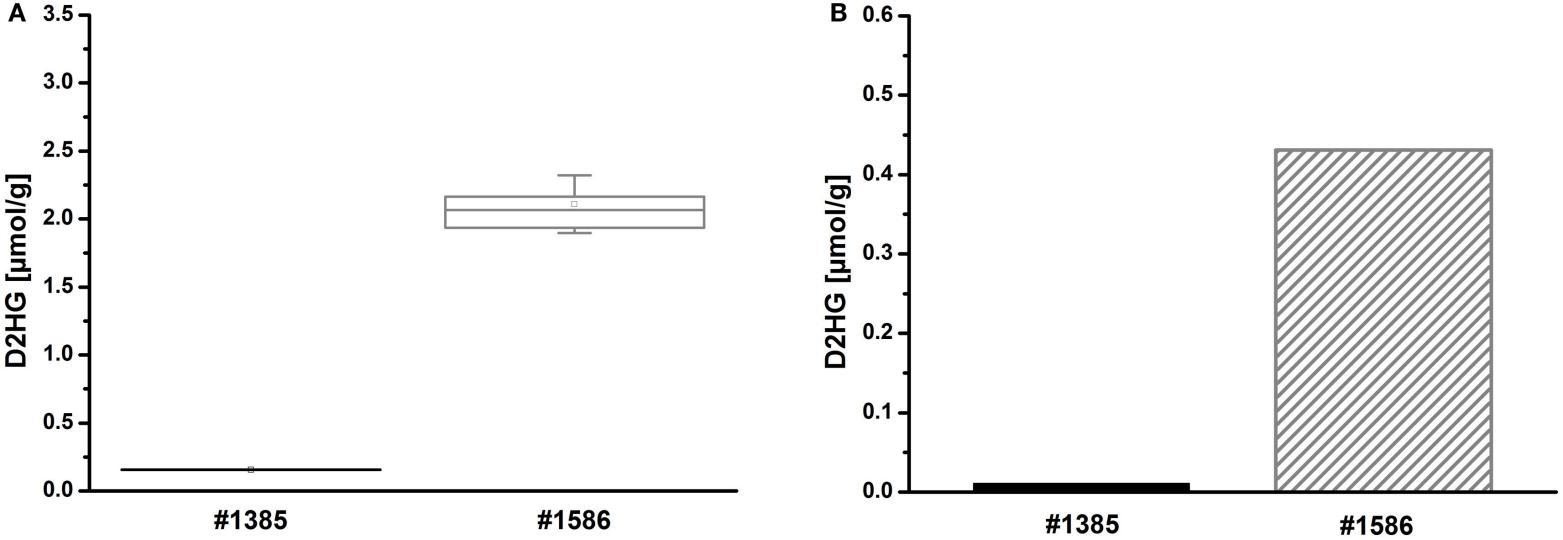
**(A)** Imaging bioluminescence of D2HG in two WHO grade IV glioblastomas of initially unknown D2HG status. Mean values from tri-/quadruplicate measurements ± SD are shown for two biopsies, one containing low *(#1385)* and the other containing high levels of D2HG *(#1586)*, respectively. Boxplot: mean *(square)*, median *(line)*, box (25 and 75% percentile), SD *(whisker)*. **(B)** Corresponding mean values ± SD from triplicate measurements of 2HG by means of achiral LC-MS/MS using internal calibration via analyte/stable isotope ratio.

Tissue sections from human glioblastoma multiforme biopsies were used as tissues containing an unknown amount of D2HG to test our new bioluminescence imaging assay for the detection of D2HG. As depicted in Figure 6, serial sections from rapidly frozen tissue revealed a D2HG distribution (Figure 6D) that was remarkably similar to that of lactate (Figure 6C) concerning the color-coded tissue distribution (the regional overlays especially) (Figure 6E) in an adjacent section. Although regarding the absolute counts, there was a marked difference between the D2HG and the lactate concentrations. In addition, bioluminescently determined concentrations of D2HG in two WHO grade IV glioblastoma biopsies were determined by means of LC-MS/MS using internal calibration via analyte/stable isotope ratio. Therefore, samples from the same biopsies (#1385 containing low D2HG amounts; #1586 containing high D2HG amounts) were analyzed in parallel via D2HG-dependent imBI and LC-MS/MS for their D2HG amount. As depicted in Figures 7A,B, the amounts of D2HG detected by imBI of D2HG and LC-MS/MS agreed, with one of the specimens (#1385) containing a low level of D2HG (0.15 ± 0.08 μmol/g via D2HG imBI; 0.01 μmol/g via LC-MS/MS), while the other (#1586) contained a comparatively high amount of D2HG (2.10 ± 0.80 μmol/g via imBI; 0.43 μmol/g via LC-MS/MS). Not unexpectedly, the absolute amounts of D2HG differ between the two techniques by 93.4% for the specimens containing low amounts of D2HG and by 79.6% for the specimens containing high amounts of D2HG. The results clearly demonstrated that these two biopsies contained largely different D2HG concentrations, which could be confirmed by LC-MS/MS. Obviously, a larger number of comparative measurements will be required to prove the reliability of the assay, but our preliminary data clearly demonstrates the great potential of bioluminescence imaging of D2HG and related metabolites in a tissue context.

## Discussion

Induced metabolic bioluminescence imaging allows visualization of endogenous metabolite distributions in cryopreserved tissues. The method has been successfully applied among others to soy bean seedlings (45), 3D cell cultures (46), various organs, surgical wounds in laboratory animals (39), and a variety of experimental tumors and human tumor tissues (43). Currently, imBI is used for the detection of ATP, glucose, lactate, pyruvate, sucrose, and glycogen (42, 43, 45, 47). With the help of the purified D2HGDH enzyme as described in Section “D2HG-Dependent Bioluminescence” (enzyme solution) in the Section “Material and Methods,” Balss et al. have previously developed a fluorimetric assay in which D2-HGDH catalyzes the oxidation of D2HG to α-KG generating NADH + H^+^ in the process. This assay showed high accordance with LC/MS measurements (35).

Here, we have adapted this assay to the special requirements of our bioluminescence imaging technique and expanded its linear range to 10 mM in order to accommodate the millimolar concentrations of D2HG that can be found in *IDH*-mutant tumors (12, 18, 48). Furthermore, we could demonstrate that the D2HG molecule is insensitive to heating in the measurement range (Figure 2), which is required to inactivate endogenous enzymes that might confound imBI. In the course of optimizing imBI for the detection of D2HG, we also observed that an increase in enzyme concentration to 5 μg/μL and a concomitant reduction of the customary assay temperature of 37–20°C (Figure 5B) increased the reproducibility of multiple measurements of D2HG levels. Furthermore, we could show that the intensity of light emitted depended not only on the tissue content of D2HG, but also on the thickness of the cryosection (Figure 6A), underscoring the known importance of preparing cryosections of equal thickness for measurement and calibration to obtain light emission that is proportional to the D2HG concentration in the tissue section (37). With the optimized assay, it thus became possible to determine *in situ* D2HG concentrations in frozen tissue sections up to 10 μmol/g tissue (wet weight).

Although designed as a study of feasibility, these preliminary results indicate that both techniques, LC-MS/MS and imBI, may identify corresponding tumors as high and low D2HG cancers, respectively. It is evident, however, that the analysis of a larger number of specimens is required to validate the novel technique with regard to its accurateness, reproducibility, and sensitivity on solid statistical grounds.

Further investigations will be needed to compare D2HG concentrations in normal and tumor tissues in a systematic way. A combination of bioluminescence imaging of ATP, lactate, glucose, pyruvate (37, 42), and D2HG will enable the characterization of the metabolic milieu of malignant and normal tissues more comprehensively in the future. As a further line of interest, the detection of elevated D2HG concentrations in different tumor entities may identify elevated D2HG levels as a surrogate marker, eventually in combination with the distribution of lactate, for tumor-relevant *IDH1* and *IDH2* mutations (3). Furthermore, D2HG detection might be of great interest in the development of selective inhibitors of mutant IDH1 and IDH2. In some neoplasms, such as AML, monitoring of D2HG might be an important parameter for remission or relapse of the disease (49). Additionally, D2HG from mutant *IDH1* and *IDH2* has been reported to alter several metabolic pathways, which could be shown in an *in vitro* overexpression system. It could be demonstrated that levels of amino acids, glutathione metabolites, choline derivatives, and TCA cycle intermediates were altered in mutant IDH1- and IDH2-expressing cells (50). Additionally, it has been demonstrated that 2-HG concentrations in serum specimens collected from patients with gliomas do not necessarily correlate with *IDH1/2* mutation status or tumor size (51). Thus, *in situ* determination of D2HG in biopsies or non-invasively by magnetic resonance spectroscopy is still required for the screening of *IDH*-mutant tumors that might benefit from alternative treatment regimens (21). Additionally, D2HG imaging may be a useful to distinguish between true tumor progression and treatment-related pseudoprogression in *IDH*-mutant gliomas (17).

Our newly established D2HG imBI method has the edge over the current standard methods applying IDH1R132H antibody staining and sequencing the IDH region, since with our method it is possible to detect all IDH mutations that can produce 2-HG and this is, additionally, possible with a spatial resolution that is otherwise only possible by the use of the IDH1R132H antibody.

In conclusion, we were able to develop a sensitive bioluminescence imaging assay for the *in situ* detection of D2HG in solid normal or tumor tissue of animal or human origin, thus providing a better insight into the distribution and effects of this oncometabolite in a tissue context.

## Author Contributions

Conceived and designed the experiments: NV, SW, and WM-K. Performed the experiments: NV, MP, KD, and SP. Analyzed the data: NV, MP, KD, SW, and WM-K. Contributed reagents/materials/analysis tools: MP, KD, and SP. Wrote the paper: NV, SW, and WM-K.

## Conflict of Interest Statement

The authors declare that the research was conducted in the absence of any commercial or financial relationships that could be construed as a potential conflict of interest.

## Abbreviations

2-HG, 2-hydroxyglutarate; 2-OG, 2-oxoglutarate; α-KG, alpha-ketoglutarate; AML, acute myeloid leukemia; ATP, adenosine triphosphate; D2HG, D-2-hydroxyglutarate; D2-HGDH, D-2-hydroxyglutarate dehydrogenase; EM-CCD, electron multiplying charge-coupled device; FMN, flavin mononucleotide; GC/MS, gas chromatography/mass spectrometry; HE, hematoxylin/eosin; HPLC, high performance liquid chromatography; IDH, isocitrate dehydrogenase; imBI, induced metabolic bioluminescence imaging; L2-HGDH, L-2-hydroxyglutarate dehydrogenase; LC/MS, liquid chromatography/mass spectrometry; NAD, nicotinamide adenine dinucleotide; NFO, NADH:FMN-oxidoreductase; RLU, relative light units; TCA, tricarboxylic acid; TET, ten-eleven translocation methylcytosine dioxygenase.
